# Listening to the Patient: Improving the Design and Conduct of Clinical Trials in Inflammatory Bowel Diseases

**DOI:** 10.1093/crocol/otaa011

**Published:** 2020-03-20

**Authors:** Dan Sharp, Sara Ringer, K T Park, Swati Tole, David T Rubin, Miguel Regueiro

**Affiliations:** 1 IBD patient advocate, DanSharpIBD.org; 2 IBD patient advocate, InflamedAndUntamed.org; 3 Genentech, Inc, South San Francisco, California, USA; 4 Inflammatory Bowel Disease Center, University of Chicago Medicine, Chicago, Illinois, USA; 5 Department of Gastroenterology, Hepatology, and Nutrition, Cleveland Clinic Foundation, Cleveland, Ohio, USA

To the Editors,

“Overall, [participating in a clinical trial] wasn’t a great experience, but it didn’t deter me from potentially enrolling in another trial. Although I’ve never had a real opportunity to enroll in one again—and at this point I’m not eligible for the ones I’m interested in. It’s incredibly frustrating…”

—Dan, inflammatory bowel diseases (IBD) patient and advocate, on his experience as a participant in a phase 3 trial for a biologic in the early 2000s.

“Due to my colectomy, I can’t participate in any of the trials I come across… How are we ever going to learn from or about patient groups normally excluded from trials if they’re not being studied?”

—Sara, IBD patient and advocate

Dan’s and Sara’s experiences emphasize the need to make inflammatory bowel diseases (IBD) trials more patient-centered. Because treatment options for IBD lag behind those for other chronic diseases, clinical trials are still needed to identify safe and efficacious therapies. Many currently available therapies do not meet patients’ long-term efficacy expectations, and expediting the development and approval of newer, nonimmunosuppressive therapies remains a critical need.^1–3^

Across disease states, particularly oncology, neurodegenerative diseases, and human immunodeficiency virus (HIV), the importance of the patient voice has increasingly been recognized, and patients’ insights have been implemented into clinical trial designs. It is time for a similar approach with IBD clinical trials. Here, we highlight why the patient perspective needs to be incorporated and share recommendations on how future trials can be better designed with the patient in mind (Table 1).

**TABLE 1.** Concerns in IBD Clinical Trials and Potential Solutions

Concern	Potential Solution
Receiving placebo or less effective treatment	Comparative efficacy trials; historical placebo data from completed trials; platform trials to reduce number of placebo arms
Lack of access to investigational treatment	Extension studies/access programs
Restrictive inclusion/exclusion criteria	Broadening inclusion/exclusion criteria; considering studies in underserved patient groups
Invasive end points/measures	Testing for biomarkers or validating noninvasive surrogate end points
Patient and caregiver burden	Telemedicine, at-home testing or follow-up, appropriate compensation for direct costs of trial participation
Clinical trial literacy (patients and physicians)	Improving education; facilitating conversations; incorporating clinical trial participation into discussions during patient care visits

## CURRENT STATE OF REGISTRATIONAL CLINICAL TRIALS IN IBD

Clinical trials designed for the approval of new therapies in IBD have typically been randomized, placebo-controlled studies with an induction phase followed by a maintenance phase. Historically, outcome measures were subjective and symptom-based, and study dropout rates were high.^4^ Over time there have been improvements, particularly the incorporation of more objective end points.^5–7^

**FIGURE 1.**
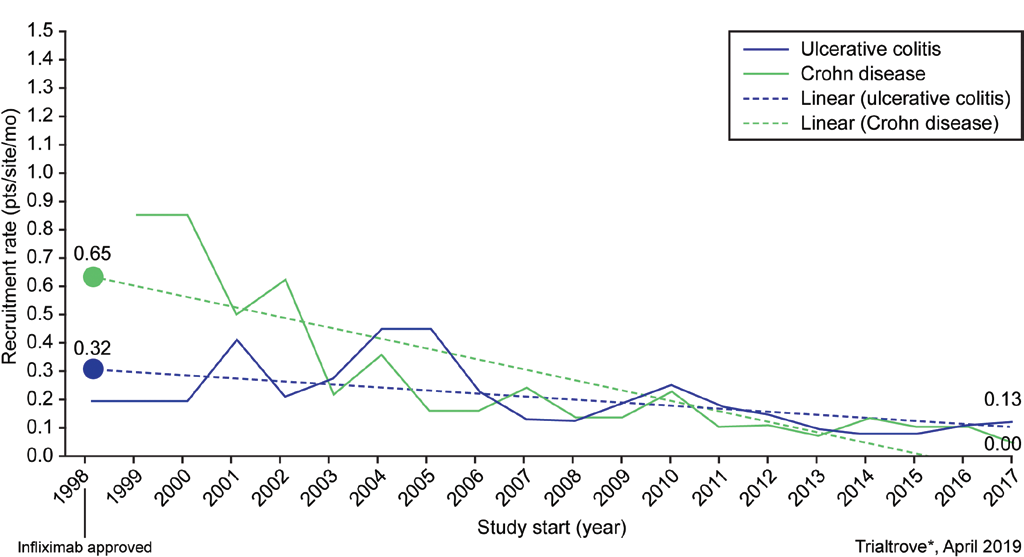
Declining recruitment rate of clinical trials in moderate-to-severe ulcerative colitis and Crohn disease.^8^ Dashed lines indicate least-squared regression fits. Source: Trialtrove | Informa, 2019. Reprinted from *Gastroenterology* 2019;157:1457–1461 with permission from Elsevier.

Despite these improvements, the extensive screening requirements and outcome assessments, use of placebo, and length of registrational trials continue to be extremely burdensome to patients. It has become increasingly difficult to enroll the large patient populations needed, making development of new therapies unsustainable.^8^ If these clinical trial practices remain the standard, this trend toward declining recruitment rates will likely continue and may worsen (Fig. 1).

Currently, IBD trials do not adequately consider the patient voice. A reflective evaluation is necessary to determine whether IBD clinical trials as they are presently conducted address the ongoing unmet medical needs of patients living with ulcerative colitis and Crohn disease.

## KEY CONCERNS IN IBD CLINICAL TRIALS

One of the major considerations in diseases with existing treatment options such as IBD is whether the comparator arm in a clinical trial should be placebo or active drug (Table 1). Despite the availability of potentially effective therapies in IBD, the majority of randomized controlled studies in ulcerative colitis and Crohn disease have required a placebo control arm (when excluding dose-comparison studies).^9^ According to the Declaration of Helsinki, the use of placebo is only acceptable if no other effective therapy exists or if no serious or irreversible harm to a patient is risked by participating in a placebo study arm.^10^ The choice of an active comparator arm ensures all patients receive treatment; however, these trials require more patients and therefore may take longer to complete than placebo-controlled trials. The potential for randomization to the placebo arm and subsequent worsening of disease, as was Dan’s experience, is a major impediment for patient enrollment in trials. On the other hand, the decision for study designs to include randomization of patients to a placebo arm should be carefully considered and use of placebo minimized when possible. Innovative approaches in trial design to limit placebo, such as the use of a historical placebo or a control arm using modeling methods, should be considered. Careful evaluation is required to ensure the utmost scientific rigor without compromising benefit/risk to the patient participating in a clinical trial.

Another potential challenge for patients is lack of access to the investigational treatment following completion of a clinical trial. This has implications for patients responding to an investigational drug during a trial; upon treatment cessation, there is the potential for loss of response or development of immunogenicity. Although many phase 3 trials include long-term extension phases to allow patients to remain on therapy until regulatory approval is attained, it may still be a problem in early phase trials, particularly if a clinical development program is discontinued.

Clinical trial eligibility criteria exclude a substantial number of patients with IBD. For example, most IBD trials require patients to have moderate-to-severe disease activity at the time of enrollment. This patient population is not representative of IBD patients in the real-world setting^11^ and may cause some patients to stop treatment before undergoing screening for a trial in order to become “sick enough” for the study, yet not so sick that they become ineligible. Due to possible safety concerns around combining therapies, trials often require washout of ongoing therapies, which can be undesirable for some patients. In addition, trials frequently exclude patients with an extensive history of failed treatments, which can be quite frustrating to patients who have no remaining treatment options. These criteria limit the opportunities for patients like Dan to enroll in other clinical trials. Inflammatory bowel diseases patient populations typically excluded from clinical trials include patients like Sara who have undergone surgery, have a history of cancer, and pediatric patients—all of whom may have unmet IBD treatment needs.

Current enrollment criteria and end points used in IBD clinical trials require invasive assessments that are associated with potential risks. Patients with IBD may experience feelings of dread prior to an endoscopy and typically must undergo more endoscopies during a clinical trial (generally 3 during a 1-year trial) than they would as part of standard care. Preliminary results from our ongoing study assessing patient perspectives on clinical trials finds that the invasive testing component of most IBD clinical trials is a major barrier to enrollment.

The patient burden of participating in clinical trials is often under-recognized. Patients and their caregivers incur costs for commuting to clinic visits and missing work and may be under-compensated for direct costs such as parking fees. Patients in a trial can be made to feel more like a “subject” than a “patient,” or like “guinea pigs,” and may be dissatisfied with the level of care they receive as part of a clinical trial. This is especially true when they must undergo invasive endoscopies as clinical trial assessments.

Finally, many patients—and some physicians—do not fully understand the clinical trial phases and the objectives of each phase, which presents another major challenge to successful conduct of clinical trials in IBD.

## HOW TO IMPROVE TRIALS FOR PATIENTS WITH IBD

Numerous opportunities exist to make meaningful, patient-centered changes to improve the patient experience in IBD (Table 1). These changes could have notable impacts, including increasing clinical trial enrollment and hastening the approval of much-needed new therapies.

Use of placebo could be minimized or eliminated by performing platform trials that rely on a single placebo control arm for multiple treatments or by performing head-to-head comparative efficacy trials. Providing patients the option of continuing treatment following completion of the study via extension studies or access programs could alleviate concerns associated with treatment cessation.

Broadening trial inclusion/exclusion criteria could reverse declining recruitment rates in IBD clinical trials and begin to address unmet needs in underserved IBD patient populations. Such populations include patients with clinically less active disease, those with a history of gastrointestinal surgery or cancer, adolescent patients, and patients currently receiving or previously treated with other IBD therapies. These patients, many of whom have complex disease, have typically been excluded from clinical trials to reduce confounding variables and maximize potential treatment effects. Including these populations may require more innovative trial designs than the traditional placebo-controlled trial, but such studies could expand patient enrollment and improve applicability of results to wider IBD populations.^11^

Within trials, validating and incorporating noninvasive surrogate end points that measure inflammation, such as biomarkers, into trial design could improve patients’ experience and potentially increase participation in clinical trials. Telemedicine, currently used in the community setting, could improve patient convenience and comfort by limiting the need for clinic visits—bringing the trial to the patient. Trials could be conducted at home, at the workplace, or elsewhere at the convenience of the patient rather than asking the patient to do what is most convenient for the trial. This would require regulatory guidance and systemic changes to trial design using technology in a patient-centered way.

Changes such as provision of travel stipends, offering food at clinic visits, and ensuring a high level of care throughout studies can help to reduce the burden of participation in trials. Educational efforts targeting both patients and physicians could help improve clinical trial literacy, clarifying the objectives of each clinical trial phase and facilitating more informed decisions about patients’ IBD care. Educational resources, such as those provided during the informed consent process, increasingly take advantage of electronic platforms to introduce interactivity and provide customizable options.^12^ Use of educational “e-portals” or support services and patient navigators, including nonclinical lay navigators, also appears to be a promising approach for improving patients’ clinical trial understanding and enrollment.^13^

## DRAWING INSPIRATION FROM OTHER DISEASES

Patient advocates in other therapeutic areas such as oncology and HIV have greatly impacted clinical trial design. HIV/acquired immunodeficiency syndrome activism led to the formation of national and local community advisory boards in 1990 within National Institutes of Health-funded clinical trial networks, providing advocates a defined role in the design, implementation, and evaluation of clinical trials.^14^ Cancer patient advocates participate in concept and protocol development for phase 1, 2, and 3 oncology clinical trials, providing feedback on eligibility criteria, study procedures, study approval, safety and confidentiality issues, and the ability to attract and retain participants.^15^ In one case, feedback from patient advocates resulted in a study reducing the number of annual endometrial biopsies from 4 to 1, a change likely to improve the patient experience as well as the likelihood of study completion.^15^

Patient advocacy groups in IBD, such as the Crohn’s & Colitis Foundation and the European Federation of Crohn’s & Ulcerative Colitis Associations, are already playing an important role in influencing the design of future IBD clinical trials. In Europe, country-specific patient advocacy groups are helping shape the global conversation toward more patient-friendly trial designs. Collaborative research initiatives between industry partners are also in consideration. In sum, efforts are currently underway to integrate input from patient advocates with that of health care providers, regulators, payers, and industry partners.^16^

The quest for precision medicine is an ultimate goal for clinical trials, and initiating the “right drug for the right patient” would be novel to IBD. Oncology has incorporated precision medicine into trial design by utilizing biomarkers that predict response to a certain medication.^17^ This could result in fewer patients required in trials, with better outcomes.

## CONCLUSION

There is an urgent need for new treatments for IBD; however, patient recruitment and enrollment in clinical trials have been a significant barrier, resulting in delays in trial completion.^8^ Incorporating the patient perspective into clinical trials and clinical care should be the new standard by which future IBD therapies are developed. For many patients, the choice to participate in clinical trials comes not only from seeking access to new treatments for themselves, but also from the desire to contribute to the development of future, more effective treatments for others with IBD. Our goal is to facilitate such participation while decreasing the burden of participation.

Integration of the patient voice in trial design as well the use of telehealth and alternative trial designs will allow a shift towards more patient-centric clinical trials in IBD. Suggestions for easier-to-implement changes to trial design are provided; however, more systemic changes to trial structure will require industry partnership, input from regulatory agencies across the globe, strategic advocacy from physicians and advocacy groups, and improved communication among all stakeholders.

## Notes

Funding: Medical writing assistance was provided by ApotheCom (San Francisco, CA) and was funded by Genentech, Inc, a member of the Roche Group.

Conflicts of interest: D.S. and S.R. declare no conflicts of interest. K.T.P. and S.T. are full-time employees of Genentech, Inc. and shareholders in the Roche Group. D.R. has received grants from AbbVie, Genentech/Roche, Janssen Pharmaceuticals, Prometheus Laboratories, Shire, Takeda, UCB Pharma; personal fees from AbbVie, Abgenomics, Allergan, Inc., Biomica, Boehringer Ingelheim, Ltd., Bristol-Myers Squibb, Celgene Corp/Syneos, Dizal Pharmaceuticals, Genentech/Roche, Gilead Sciences, Glenmark Pharmaceuticals, GSK (GlaxoSmithKline Services), Janssen Pharmaceuticals, Lilly, Mahana Therapeutics, Narrow River Management, Pfizer, Prometheus Laboratories, Reistone, Seres Therapeutics, Shire, Takeda, Target PharmaSolutions, Inc., Techlab, Inc., GalenPharma/Atlantica, Medtronic, Arena Pharmaceuticals, Ferring Pharmaceuticals, Inc., Merck & Co., Inc., Napo Pharmaceuticals, Amgen, Forward Pharma, Salix Pharmaceuticals, Inc., Miraca Life Sciences, Mitsubishi Tanabe Pharma Development America, Inc., Samsung Bioepis, Sandoz Pharmaceuticals. M.R. has received research support from AbbVie, Janssen, Takeda, Pfizer; unrestricted educational grants from AbbVie, Janssen, UCB, Pfizer, Takeda, Salix, Shire; has consulted and/or participated in advisory boards for AbbVie, Janssen, UCB, Takeda, Pfizer, Miraca Labs, Amgen, Celgene, Seres, Allergan, Genentech, Gilead, Salix, and Prometheus.
